# High incidence of adrenal suppression in children with Kawasaki disease treated with intravenous immunoglobulin plus prednisolone

**DOI:** 10.1186/1687-9856-2015-S1-P39

**Published:** 2015-04-28

**Authors:** Masahiro Goto, Naoyuki Miyagawa, Kaori Kikunaga, Masaru Miura, Yukihiro Hasegawa

**Affiliations:** 1 Tokyo Metropolitan Children’s Medical Center Fuchu, Japan; 2 Kanagawa Children’s Medical Center, Yokohama, Japan

## Context

Combination treatment with intravenous immunoglobulin (IVIG) plus prednisolone, newly designed for children with severe Kawasaki disease (KD), significantly reduces coronary artery abnormalities [1]. Prednisolone is administered for approximately 20 days in this regimen.

## Objective

Our aim was to examine whether adrenal function of the treated patients is suppressed by glucocorticoid administration in this regimen.

## Design/setting

This was a prospective study at one medical institution.

## Patients

We analyzed data from 21 children with KD who were treated with IVIG plus prednisolone between February and June, 2012.

## Main outcome measures

The main outcome measures were cortisol and ACTH values in the morning after the cessation of prednisolone administration and peak cortisol and ACTH values at CRH stimulation tests repeated 0, 2, and 6 months after the treatment.

## Results

Morning cortisol and ACTH values after the cessation of prednisolone treatment were suppressed. Peak cortisol values at the first CRH stimulation test ranged from 5.1 to 25.4 mcg/dL and were less than 20 mcg/dL in 17 of 21 patients, but were restored to more than 14.6 mcg/dL in all of them by 6 months after the prednisolone treatment. A significant positive correlation was observed between cortisol values at 09:00 after the prednisolone treatment and peak cortisol values at the following CRH stimulation test (r = 0.770, p < 0.0001).

## Conclusions

Adrenal suppression can occur in a high proportion of children with KD treated with IVIG plus prednisolone, despite rather short duration and low administered dose of glucocorticoids.
